# Editorial: Mass spectrometry-based proteomics in drug discovery and development

**DOI:** 10.3389/fmed.2024.1448152

**Published:** 2024-07-23

**Authors:** Martin Pejchinovski, Pedro Magalhães, Jochen Metzger

**Affiliations:** ^1^Department of Analytical Instruments Group, Thermo Fisher Scientific, Germering, Germany; ^2^VIB KU Leuven Center for Brain & Disease Research, Leuven, Belgium; ^3^Medical Health Services/In Vitro Diagnostics, TÜV SÜD Product Service GmbH, Hamburg, Germany

**Keywords:** mass spectrometry, proteomics, biomarker, clinical application, drug development

Mass spectrometry (MS) has become an important analytical tool, and it plays a pivotal role in the identification and characterization of proteins and peptides for clinical applications. Continued technological development in combination with recent advances in data analysis allows the progression toward faster and deeper proteome coverages, and this has enabled the long-awaited step forward of analyzing thousands of proteins in one sample with high sensitivity and in a highly standardized manner. Together with recent advances in clinical medicine, these new proteomics strategies have enabled a precise description of the complex molecular and systems biology interactions in the field of biomarker research and biomarker-driven drug development (1, 2).

The path from early proteomic biomarker discovery to more meaningful clinical application is challenging and still requires further technical achievements to fully exploit the potential of MS technology. Most strikingly, this is highlighted by recent publications on the use of MS for protein expression analysis and the complete mapping of post-translational modifications depending on high-throughput technology for full sequence coverage (3, 4). On the other hand, those performing biomarker-driven clinical trials show an ever-growing interest in capturing and investigating entire molecular features and understanding their interplay and regulation. The reason for such an endeavor is obvious: it can serve as a key to individualized treatment therapies well founded on a proteomics-based patient stratification. However, the clinical utility of such an approach must also consider patient safety and comply with ethical, legal, and regulatory requirements (3–5).

The aim of the present Editorial, entitled “*Mass spectrometry-based proteomics in drug discovery and development*,” is to highlight the importance of MS-based biomarker discovery for clinical applications. By doing so, it is our utmost interest to present the reader of Frontiers in Medicine some recent advances in this field to make clear how MS biomarker analysis can be effectively implemented in clinical trials. The selected examples are not limited to diagnosis, prognosis, and treatment monitoring but extend the use of MS biomarker analysis to drug discovery and lead development. In the first article by Kwon et al., the authors provide a comprehensive overview of current trends in using multi-omics data integration for diagnostic purposes and for the development of new treatment strategies. For practical implementation of such an approach, the authors refer to several promising studies. One of these explores the role of pyrroline-5-carboxylate reductase (PYCR) and alcohol dehydrogenase 1A (ADHA1) as prognostic biomarkers for tumor growth in the setting of hepatocellular carcinoma (HCC) and how these biomarkers can serve as targets for the development of a new treatment regimen. Other examples of cancer biomarkers include liver kinase B1 (LKB1), known as a tumor suppressor gene, which was demonstrated by MS to be involved in the regulation of the m-TOR-dependent pathway and the growth of pancreatic cells; cancer-associated fibroblast (CAF), another metabolic modulator, was linked to enhancement in ovarian cancer. Finally, it describes, besides several other alternations in protein expression patterns, the identification of transcription regulator proteins and CHC homology 1 (BACH1) as important contributors to metastasis in Glioblastoma multiforme (GMB), a very aggressive primary brain tumor.

Included in this series are two manuscripts that provide new insights into breast cancer. In the first article, Yao et al. explored the proteomic signature of adenoid cystic carcinoma (ACC), a rare type of triple-negative breast cancer. Given the similarities in morphology, immunohistochemistry, and molecular signatures with basal-like triple-negative breast cancer (BL-TNBC), accurate differentiation of these two neoplasms is crucial for effective clinical treatment and targeted therapy of ACC. Despite the limited number of patients, the comparative proteomic and clinicopathological analysis between ACC and BL-TNBC points toward a suppression of the immune response and vesicle-mediated transport, while it showed increased activity in extracellular matrix organization, ribosome biogenesis, and RNA splicing. In addition, overexpression of Integrin beta 4 (ITGB4), Versican (VCAN), and Dermatopontin (DPT) have been identified as ACC biomarkers and were proposed by the authors as promising targets for ACC-directed therapy.

In the second article focused on breast cancer by Xu et al., targeted metabolomics profiling by MS was applied to unravel the metabolomic signature of breast cancer in blood plasma. A well-characterized cohort of 229 patients with either benign breast conditions or malignant breast cancer was included in the study to establish a diagnostic classifier and evaluate its predictive value. As was demonstrated by the authors, 716 metabolites showed statistical differences and were qualified in subsequent confirmatory examinations. By applying various statistical and pathway enrichment methods, the serotonergic synapse pathway was identified to be differently regulated between the benign and malignant patient groups. This is in line with previous findings that neurotransmitters derived from this pathway are significantly involved in breast cancer pathophysiology. Moreover, the authors combined 14 metabolites into a diagnostic classifier for better decision-making in clinical practice; this must, however, be further validated in clinical practice.

As an example, for the use of MS-based proteomics in animal models, the article by Jentsch et al. was selected. It describes the identification of new biomarker candidates in severe equine asthma (SEA), by utilizing immunoproteomics in combination with MS. SEA is the equine counterpart to neutrophilic asthma in humans. The authors first determined the proteome profiles of Aspergillus fumigatus, a common mold species in hay known as the major cause of SEA. The authors identified for several of these A. fumigatus allergens significant differences in Ig binding between asthmatic and healthy horses. Four A. fumigatus allergen candidates could be confirmed as Ig antigens after their recombinant expression. These are beta-hexosaminidase, class II aldolase/adducing domain protein, glucoamylase, and the peptide hydrolase B0XX53. The identified A. fumigatus allergens might serve as the basis for future studies on the molecular mechanisms of SEA and as protein targets for the development of new diagnostics and therapeutics approaches.

In recent years, the study of proteomics by use of mass spectrometry techniques has revolutionized our understanding of the molecular biology of disease processes. The utilization of proteins and peptides for clinical applications has steadily grown in relevance and impact (6). In fact, it is well known that disease processes and drug effects have a tremendous impact on the proteomic composition of cells, tissues, and body fluids. Attributed to their highly dynamic regulation in response to hemostatic changes or environmental stimuli, proteins are the most ideal targets to detect and monitor disease processes and therapy effects on the molecular level Unraveling the role of proteins in disease mechanisms will pave the way to protein biomarker-directed targeted therapy and potentially in the future to protein-based therapeutics (6, 7).

## Author contributions

MP: Conceptualization, Investigation, Methodology, Supervision, Writing – original draft, Writing – review & editing. PM: Writing – original draft, Writing – review & editing. JM: Writing – original draft, Writing – review & editing.
